# The Boom in 3D-Printed Sensor Technology

**DOI:** 10.3390/s17051166

**Published:** 2017-05-19

**Authors:** Yuanyuan Xu, Xiaoyue Wu, Xiao Guo, Bin Kong, Min Zhang, Xiang Qian, Shengli Mi, Wei Sun

**Affiliations:** 1 Tsinghua-Berkeley Shenzhen Institute, Shenzhen 518055, China; xuyy16@sz.tsinghua.edu.cn (Y.X.); lingxue0313@163.com (B.K.); 2 Graduate School at Shenzhen, Tsinghua University, Shenzhen 518055, China; wuxiaoyu14@mails.tsinghua.edu.cn (X.W.); guoxiao14@mails.tsinghua.edu.cn (X.G.); zhang.min@sz.tsinghua.edu.cn (M.Z.); qian.xiang@sz.tsinghua.edu.cn (X.Q.); 3 Open Fiesta, Tsinghua University, Shenzhen 518055, China; 4 Department of Mechanical Engineering, Drexel University, Philadelphia, PA 19104, USA; 5 Department of Mechanical Engineering and Mechanics, Tsinghua University, Beijing 100084, China

**Keywords:** 3D printing, sensors, additive manufacturing

## Abstract

Future sensing applications will include high-performance features, such as toxin detection, real-time monitoring of physiological events, advanced diagnostics, and connected feedback. However, such multi-functional sensors require advancements in sensitivity, specificity, and throughput with the simultaneous delivery of multiple detection in a short time. Recent advances in 3D printing and electronics have brought us closer to sensors with multiplex advantages, and additive manufacturing approaches offer a new scope for sensor fabrication. To this end, we review the recent advances in 3D-printed cutting-edge sensors. These achievements demonstrate the successful application of 3D-printing technology in sensor fabrication, and the selected studies deeply explore the potential for creating sensors with higher performance. Further development of multi-process 3D printing is expected to expand future sensor utility and availability.

## 1. Introduction

Three-dimensional (3D) printing, known as additive manufacturing, has attracted much attention from the public and the media in recent years and describes a family of techniques that involve the fabrication of 3D components using material jetting, powder bed fusion, material extrusion, sheet lamination, directed energy deposition, photopolymerization, and binder jetting. These methods create components in a layer-by-layer manner and offer various options regarding cost, feature details and materials. The most popular materials are polymers, metals, composites and ceramics. 3D printing enables the creation of complex geometric shapes and merging of selected functional components into any configuration, thus supplying a new approach for the fabrication of multifunctional end-use devices that can potentially combine optical, chemical, electronic, electromagnetic, fluidic, thermal and acoustic features.

A sensor is defined as an object that detects events or changes in the environment and sends the corresponding real-world data to the computer. With the development of micro-machinery and advances in micro-controller platforms, elaborate sensors have been widely applied in manufacturing and machinery, aerospace and airplanes, medicine and biomedical devices, and robotics. 

Increasing interest has focused on the use of 3D-printing technology for the manufacturing of sensors. The 3D-printing process can be started and stopped to incorporate complementary fabrication processes or to embed subcomponents manufactured using traditional methods. Thus, the process of 3D-printed sensors fabricated by either embedding a sensor into printed structures or intrinsically printing the entire sensor can be conducted seamlessly [1]. In recent years, a considerable amount of current research on 3D-printed sensors has focused on selected areas such as electronics, force, motion, hearing, optics, etc. Electronic and force sensing modules are particularly well suited for 3D printing, and other sensing categories tend to be manufactured by the integration of commercial components into 3D-printed structures. Printed sensors combine several important technologies, such as printing technology and electronic device design. Electronic devices manufactured by various printing processes for customized substrates are the key components and main trends of 3D-printed sensors. 3D-printed sensors should include the following key components: substrate board, electronic ink, and print processing technique [2]. Typical conductive inks contain carbon conductive ink, polymer conductive ink, nano-silver ink and liquid metal ink. The concept of printed electronics was initially proposed in the early 1990s. In 1994, Francis Garnier realized an all-polymer field-effect transistor using printing techniques [3]. Bao and Feng used a screen-printing technology to produce a transistor for the first time [4]. Jacobson and colleagues printed an organic transistor in 1999 [5]. Inkjet-printed circuits were demonstrated by Sirringhaus and colleagues in 2000 [6]. The manufacture of these basic electronic devices using 3D-printing technology offers a new approaches for the fabrication of sensors. Advancements in multi-process and hybrid 3D printing are leading to the fabrication of sensors that are both geometrically complex and functionally complex and are easily assembled.

In this review, we first briefly introduce the main techniques of 3D printing (some key characteristics are discussed and summarized in Table 1) and then provide an overview of current 3D-printed sensors, discuss the advances and limitations in the different fabricating processes, and then describe a number of investigated devices according to their categorization by application. 

The innumerable intelligent designs provided by 3D printing technology cannot be exhaustively covered in this review of all applications, and rather the examples included here are intended to highlight the potential of the technology and motivate the further advances. The detailed information of these examples, including 3D printing technologies, transduction mechanism, application, printing materials, and 3D-printed parts are listed in Table 2.

## 2. A Brief Review of 3D Printing

**Figure 1:**
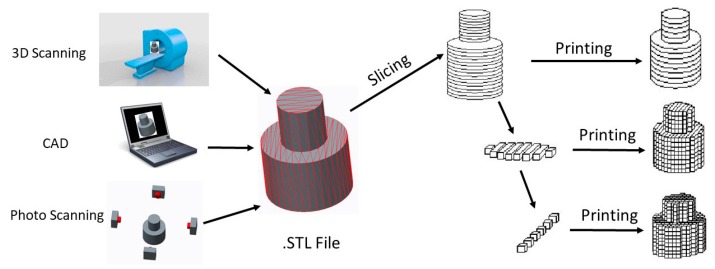
The process of 3D-printing.

A 3D-printing process starts with a digital model of the object to be printed. The virtual model can be achieved using a three-dimensional scanner (like CT), computer-aided design (CAD) software, or by making use of photogrammetry technology which obtains the model through the combination of images of the object obtained by a photo scanning process performed from different positions. The 3D model needs to be converted into an STL file after creation. This STL file contains a list of coordinates of triangulated sections which store the information about the model’s surfaces. All 3D printer software can read STL files, and then slice the object to obtain a series of 2D cross section layers by a Z direction discrete approach. Finally, the desired 3D object is created using layer by layer printing. A specific 3D printing process is shown in Figure 1. Depending on the manufacturing principles, 3D printing technologies applied in the fabrication of sensors can be divided into seven main categories: fused deposition modeling (FDM), directly ink writing (DIW), photocuring (SLA, DLP), lamination (LOM), laser sintering and laser melting (SLS, SLM), photopolymer jetting (Ployjet) and binder jetting (3DP). In hybrid 3D printing process, models are fabricated using a combination of traditional manufacturing methods and the additive manufacturing methods mentioned above.

### 2.1. Fused Deposition Modeling (FDM)

**Figure 2:**
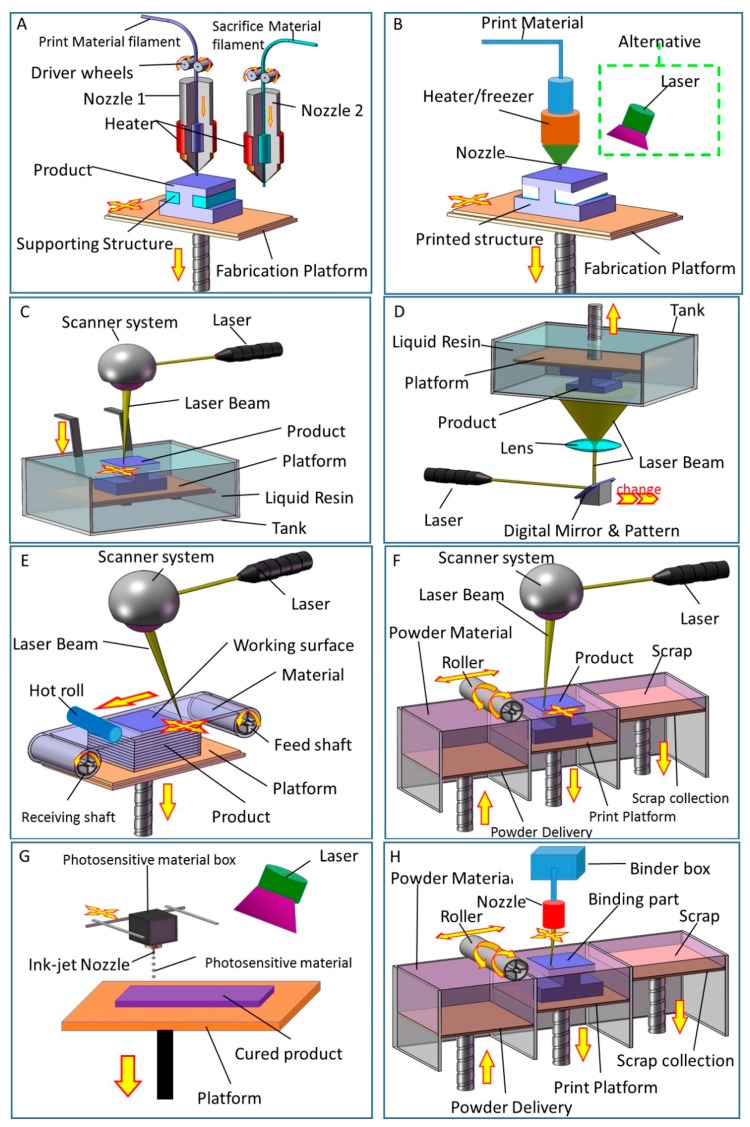
3D printing technologies. (**A**) Fused deposition modeling (FDM); (**B**) Directly ink writing (DIW); (**C**) Stereolithography (SLA); (**D**) Digital light procession (DLP); (**E**) Lamination (LOM); (**F**) Selective laser sintering (SLS) and Selective laser melting (SLM); (**G**) Photopolymer jetting (Ployjet); (**H**) Binder jetting(3DP).

FDM was first introduced by Crump [61]. FDM 3D printers’ working principle involves melting and extruding a thermoplastic filament through a nozzle (Figure 2A). The melted material deposited on the fabrication platform then cools down and solidifies, and this this process is repeated in a layer-by-layer fashion to build up a 3D structure. Thermoplastic materials such as polyamide (PA), polylactic acid (PLA), acrylonitrile butadiene styrene (ABS), polycarbonate (PC), etc. are usually employed and provided as a filament for FDM 3D printers. FDM has been widely used for its low material cost and open source nature, but it is limited by its low printing resolution and slow printing speed.

### 2.2. Direct Ink Writing (DIW)

Direct ink writing printers use nozzles that directly extrude materials onto a fabrication platform (Figure 2B). This technology allows the controlled deposition of materials in a highly viscous liquid state, which allows them to retain their shape after deposition. Direct ink writing technology is extremely versatile because a large variety of materials can be deposited, ranging from ceramics, plastics, foods, hydrogels and even living cells [62,63]. The nozzle size, viscosity and density of the material, scanning speed, eject speed and other parameters can be adjusted to obtain an optimal deposition object. A post-fabrication process may be need to harden the created object and improve its mechanical properties via sintering, heating, UV curing and drying steps.

### 2.3. Photocuring (SLA, DLP)

Photocuring uses ultraviolet (UV) light to cure liquid polymers in a layer-by-layer manner, building 3D structures on the platform. There are two types of photocuring technologies: stereo lithography apparatus (SLA) [64] and digital light processing (DLP) [65].

Figure 2c shows the fundamental principle of SLA. A tank is filled with a liquid photosensitive resin, which changes from liquid to solid when exposed to a certain ultraviolet light wavelength. The laser scanning of the layered cross section under the control of the computer leaves the layer cured. The cured layer is covered with a layer of liquid resin after the platform reduces the height of a layer. [66]. Then a new layer is ready to be scanned, and the new cured layer is firmly glued on the preceding layer. The steps above are repeated until all the parts of the digital model are completed, and a 3D model is obtained [67]. SLA cures the photosensitive resin by means of a moving laser directly, whereas DLP uses a laser or UV lamp as the light source. The light shines through special patterns on a digital mirror device, then the exposed parts are cured and a layer is finished. The platform rises a height of a layer and the next exposure period starts. A 3D solid model is obtained when all the layers have been exposed to the light [68]. Figure 2D shows the fundamental principle of DLP. The digital mirror device used as a dynamic mask is the main difference between SLA and DLP. SLA and DLP can produce highly accurate structures with complex internal features, but have the disadvantage of being limited to the use of a single-material.

### 2.4. Lamination (LOM)

Laminated object manufacturing (LOM) [69] uses lasers or knives to cut sheet materials. When a layer is cut, another sheet is added. The new layer can be firmly adhered to the completed parts by a roller that compacts and heats/glues the sheets together. The above steps are repeated until the process is completed. Finally, a 3D solid model is finished after removing the useless sections [70]. Figure 2E shows the fundamental basis of LOM

### 2.5. Selective Laser Sintering and Selective Laser Melting (SLS, SLM)

Selective Laser Sintering (SLS) [71] or Selective Laser Melting (SLM) [72], use powdery materials, mainly including plastics, metals, ceramics, and waxes. A layer of powder is laid on the workbench. A high-strength laser is used to scan the profile to melt and coat a layer of powder onto a fabrication platform (Figure 2F). Following the sintering of one layer, the fabrication platform is lowered and the powder is tiled on top of the previous layer before sintering the next layer. By repeating this process, the layers of the 3D structure are built up on the fabrication platform. SLA and SLM technologies can print things with high enough strength and density to meet aerospace or military standards.

### 2.6. Photopolymer Jetting (Ployjet)

Photopolymer jetting was originally introduced by Gothait [73]. For Ployjet, a photosensitive resin is used as printing material. This photosensitive resin is ejected from an inkjet nozzle and deposited on a mobile platform, then cured by UV light and solidified (Figure 2G). This approach allows layer-by-layer fabrication. A 3D product can be obtained after curing all layers of the entire model. This method can print products with multiple materials and colors simultaneously. Ployjet is suitable for printing small and delicate objects due to its high-resolution. The strength of parts produced by this process is however weak.

### 2.7. Binder Jetting (3DP)

In this technique special adhesives are ejected from an inkjet nozzle and deposited onto thin layers of powder. This process bonds the layer of powder materials and produces a solid structure. When repeated, a 3D structure can be built up layer-by-layer on the print platform (Figure 2H). This approach does not need any support structures as the powders can support themselves. 3DP printers can work with a variety of powder materials, such as ceramics, plaster and sugar. This technology can print multiple materials, but the strength and surface roughness of objects are not good.

## 3. Sensor Applications

### 3.1. Force Sensors

Force sensors are crucial components in a large range of devices and systems, including robot manipulators, manufacturing processes, haptic interfaces and transportation. Commercial force sensors are generally not adapted to specific system requirements, resulting in the frequent use of sensors with excessive size, cost, and fragility [74]. To overcome these issues, 3D-printing technology has been used to create functional components for the quick, inexpensive and easily customized manufacture of force sensors. Force sensors convert applied forces into electronic signals by measuring the displacement or strain of an internal structural element known as a flexure [74]. A general force sensor usually contains three components: the flexure (which converts forces applied to the sensor along a specific direction into a displacement or strain that can be measured by the transducer), a transducer (that converts the displacement into an electrical signal), and packaging (to protect the components and facilitate mechanical connections to the remainder of the system). General-purpose commercial force sensors have limitations that restrict their utility because they are usually designed to work with a wide range of systems and loading situations. The packaging elements must be designed to avoid deflection, and the mounting provisions require rigidity for the connection of the force sensor with the system. The sensing of multiple directions of force or torque often requires complicated structures to couple multiple conventional sensors, resulting in excessive size and mass compared with the system. 3D-printed force sensors offer a greater number of advantages than discrete general-purpose force sensors. A 3D-printed force sensor tailored to the configuration of the system might reduce the need for a rigid mounting interface between the system and the sensor. Three-dimensional printing technology allows force sensors to be quickly translated from a concept to a useful device. A miniature and intricate force sensor can be printed and easily adapted for specialized applications and is compatible with MRI or chemically corrosive environments [75,76].

The mechanical properties, size, and shape of the flexure determine the sensitivity, accuracy, and directional response of the sensor [74]. The stiffness of the flexures is determined by its dimensions and material properties. In general, force sensors use high-stiffness flexures to produce small maximum displacements and small strains. Strain gauges and piezoelectric elements have good linearity and higher resonant frequencies and are small strain transducers suitable for measuring small displacements and small strains. The use of small strain transducers leads to complexity and difficulty in assembly, and small measured signals also require sophisticated electronics. 3D-printed materials such as plastics are susceptible to viscoelastic and hysteretic properties and often have low yield strengths and therefore are not suitable for flexures. Metal flexures can be easily inserted into customized designed slots in 3D printed structures, which facilitates the use of excellent elastic materials in a variety of sizes and stiffness values such that the same sensor can be configured to measure different force ranges. High-elastic alloy flexures can be used to increase the displacement range of the sensor for a given force or an overload force [77].

Conventional force sensors use strain gauges or piezoelectric transducers to create an output signal related to the applied loading [78]. In general, these devices require complex signal conditioning and elaborate mounting techniques [79]. The ideal design for 3D printing used in sensor transducers is simple to install and is compatible with a wide range of flexure displacements and dimensions [74]. Displacement sensors are suitable for use in 3D-printed sensors when coupled with relatively large strains. Hall-effect sensors and fiber optic sensors are highly suitable for measurement technologies [76,78]. These inexpensive, simple, noncontact and large-range transducers are candidates for flexure designs and high-sensitivity applications. Researchers have developed many fiber optic transduction mechanisms, interferometric and spectrally based sensors, and intensity modulation techniques [76,80]. The transducer measures displacement by determining the amount of light reflected from a surface as it moves relative to the sensor. The fibers inserted into the force sensor are electrically, magnetically, and chemically inert. When a transducer is manufactured, fibers can be inserted into the printed structures of the force sensors. Using the motion of the magnet attached to the flexure, Hall-effect sensors can detect the displacement [81,82].

The packaging of sensors can protect the inner flexure and transducer structures and potentially creates the means for mounting to other structures. Even if manufactured by 3D printing, the packaging and mounting sensors should be stiff and resist any forces that might affect or damage the sensor. Conventional packaging should also allow for easy assembly and integration with other components of the system. If sensor packaging is directly printed with a multiple-step system or as a component of a mechanism, additional packaging or mounting components are not necessary.

#### 3.1.1. Strain Sensors

Since the advancement of materials and 3D printing, fabrication techniques have driven the development of smaller, faster, and more efficient devices [7]. Strain sensors are used to detect an electrical shift upon mechanical deformation and have found broad applications in infrastructural and automobile health monitoring [83]. Stretchable electronics is a new class of electronic devices that has the potential to offer exciting opportunities, particularly in the area of large-area electronics, and has been applied in soft robotics [84,85,86,87], wearable electronics [88,89,90,91], human-machine interfaces [92,93], and other areas [94,95,96,97,98,99,100,101,102]. In general, soft strain sensors are typically composed of a deformable conducting material patterned onto, attached to, or embedded in an inactive stretchable material. Planar-printing [103], lithographic [104], coating [105] and lamination [8] techniques can all be used to create soft strain sensors. These methods are suitable and effective for creating sensors, but limitations such as high cost, limited extensibility, poor durability and lack of scalability for manufacture have hampered their wider application. The 3D-printing approach offers new avenues for the creation of soft strain sensors. Most of the 3D-printed strain sensors are enveloped in customized shapes of soft matrices, and these soft stretchable structures are both highly conformal and extensible [106,107,108]. Nanoparticles [109], nanotubes [110], nanowires [111] and graphene [112] have been reported as promising building blocks in inks for innovative 3D-printed strain sensors with enhanced performance.

**Figure 3:**
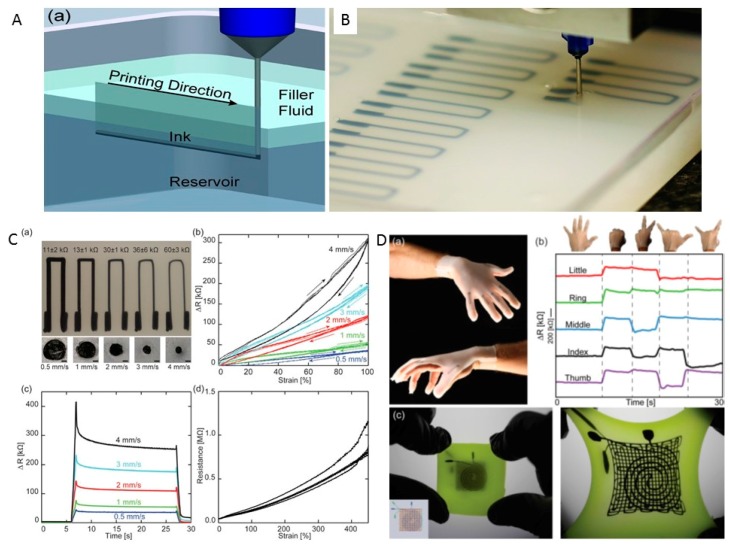
(**A**) Schematic illustration of the embedded 3D-printing process in which conductive ink is printed into an uncured elastomeric reservoir; (**B**) Photograph of e-3DP for a planar array of soft strain sensors; (**C**) Top and cross-sectional images (**a**) of soft sensors, and electrical resistance change as a function of cyclic deformation (**b**), step deformation (**c**) and mechanical failure (**d**); (**D**) Photograph of a glove with embedded strain sensors (**a**) produced by e-3DP, electrical resistance changed as a function of time for strain sensors within the glove at five different hand positions (**b**), and photograph of a three-layer strain and pressure sensor (**c**). Reproduced from [7], with permission from WILEY-VCH Verlag GmbH & Co. KGaA, Weinheim, Germany, © 2014.

Muth and coworkers [7] reported an embedded 3D-printing (e-3DP) method for fabricating strain sensors within highly conformal and extensible elastomeric matrices (Figure 3). Viscoelastic ink directly extruded into an elastomeric reservoir through a deposition nozzle forms the resistive sensing element, and the reservoir serves as the matrix material (Figure 3A,B). As the nozzle moves across the reservoir, the void space is subsequently filled by a capping (filler fluid) layer [113]. To form a monolithic component, the reservoir and filler fluid are co-cured after printing, and the embedded conductive ink remains fluid. Using this technique, soft strain sensors can be arbitrarily created in planar and 3D motif geometries in a highly programmable and seamless manner. By eliminating interfaces that give rise to delamination between individual layers, the sensor’s mechanical reliability is significantly improved. The cross-sectional dimensions of each printed sensor are controlled by adjusting the nozzle size, applied pressure, and printing speed. As an example, the strain sensors shown in Figure 3C) are printed using a fixed nozzle diameter (D = 410 μm) and applied pressure (P = 50 psi) and varying the print speed from 0.5 mm/s to 4 mm/s. Using smaller nozzles coupled with higher printing speeds, it is possible to reduce the overall dimensions of these e-3DP strain sensors. To investigate the sensor performance in cyclic strain, the embedded strain sensors described above are extended to 100% strain at a crosshead speed of 2.96 mm/s and relaxed back to a zero strain condition at the same rate. The results show that sensors with a smaller cross-sectional area produce a larger change in resistance for a given strain compared with those with larger cross-sectional areas (Figure 3C). These same researchers investigated the sensor response to a step strain input, as shown in Figure 3C(c)). The soft sensors with smaller cross-sectional areas exhibited increased sensitivity compared with those printed with larger cross-sectional areas. To evaluate the failure strain, five representative strain sensors (printed at 2 mm/s) were tested by extending them at a crosshead speed of 5 mm/s until failure. Each sensor exhibited consistent and predictable electrical response up to ~400% strain (Figure 3C(d)). The above observations reveal that the e-3DP system can readily produce mechanically robust sensors with tunable properties. For applications, printed strain sensors were created within a pre-molded, glove-shaped reservoir (Figure 3D) to produce a glove that fits on a user’s hand and monitors digit movement. The glove was used to monitor the digit motion of a user in real time (Figure 3D(b)). Using e-3DP, a three-layer sensor was produced (Figure 3D(c)) that was modeled after a biaxial strain and pressure sensor. This strain sensor is created in a single step, resulting in a fully continuous, monolithic and soft product.

Frutiger and co-workers [9] created textile-mounted, capacitive soft strain sensor (CS3) fibers for the detection of elongational strains via the multicore-shell printing approach. Each fiber contained four concentric layers, and the silicone elastomers served as the conductor, dielectric or encapsulate. The dimensions of these four-layer fibers are dictated by the nozzle sizes, respective flow rates of the ink in each layer and the printing speed. Using this multicore-shell printing-method, CS3s can be fabricated in a flexible and programmable manner. To demonstrate the utility of the fibers as a strain sensor, the sensors were mounted onto textiles, and the capacity of the fibers was tested to capture the gate cycle of a wearer in real time. The results showed that the sensors demonstrated accurate and hysteresis-free performance under both static and dynamic conditions. These customizable fiber sensors can be applied in wearable electronics, soft robotics and human/machine interfaces.

An and coworkers [10] developed a strategy for the printing of a graphene aerogel for flexible wearable electronic sensor devices. The printed structure creates a controllable 3D porous nanostructure with excellent conductivity, which is suitable for use as a multi-recognition flexible wearable electric sensor. This sensor can run multi-channel analysis for the complicated perception of movement. With rational design, the sensor can enable remarkable gesture language analysis. The device can be used as an auxiliary product for deaf-mute communication or gesture manipulation apparatus.

#### 3.1.2. Pressure Sensors

Pressure sensors have been widely applied for structural loading and gas and liquid pressure measurement. Most pressure sensors are used to measure the variation of pressure, but others are designed to measure a target pressure or for use as a switch [114]. A number of micro-electro mechanical systems (MEMS) designs have been proposed for sensing [115,116,117,118,119]. In general, the cost of these pressure sensors is high, but based on additive manufacturing, sensors can be built at low cost. The combination of 3D designs on computers and 3D printing could facilitate rapid sensor optimization.

Laszczak et al. [11] designed a capacitance-based sensor for monitoring stresses at the stump–socket interface of lower-limb amputees. This sensor delivered measured pressure (σ_p_) and shear (σ_s_) stresses. Using 3D-printing technology, the fabricated sensor had the ability to adopt the bespoke shapes of lower-limb residua under low-cost and versatile solutions. A flexible frame (20 mm × 20 mm) with a thickness of 4 mm is the main structure of the sensor, and a parallel plate capacitor that transduces mechanical deformation is the sensing mechanism. Three separate electrodes were designed on the top surface (i.e., Ex, Ey and Ez) with one bottom common electrode (Ecom) attached to the sensor. The Ex, Ey and Ez formed capacitors of Cx, Cy, and Cz paired with Ecom, respectively. The mechanical frame deforms when subjected to applied loads of pressure and shear, resulting in changes in the separation distance (d) and overlapping area (A) of the respective electrodes. These changes induce changes in the capacitors of Cx, Cy, and Cz. The test results showed that the sensor is capable of monitoring pressure and shear at stresses up to 350 kPa and 80 kPa. The sensor demonstrated high linearity (approximately 5–8%) and high performance for pressure (approximately 1.3 kPa) and shear (approximately 0.6 kPa) stress resolution. The electromechanical characteristics of the sensor derived from experiments demonstrated that the capacitive response ΔC as a function of σ_p_ and σ_s_ showed excellent agreement with the FEA prediction. Additionally, σ_p_ can be obtained directly from ΔCz, which is independent of σ_s_. σ_s_ can be obtained from ΔCx and ΔCz, ΔCx is linear with respect to σs, and the y-intercept varies with σ_p_. The influence of σ_p_ on the y-intercept of ΔCx also be obtained. The value of σ_s_ can be made independent of σ_p_ and removing the influence of σ_p_ on ΔCx allows for accurate measurements of σ_s_. All of the above results suggest that the reported sensors have strong potential in pressure and shear loading measurements at the critical stump-socket interface.

Saari et al. developed a capacitive force sensor by combining fiber encapsulation additive manufacturing (FEAM) and thermoplastic elastomer additive manufacturing (TEAM) [12]. This sensor consists of a 3D-printed rigid frame with embedded wires and a thermoplastic elastomer dielectric spacer. Wires in a tight spiral pattern emulate a flat plate capacitor, and the spacer is compressed under load force. The two electrically conductive flat regions are separated by a dielectric material that effectively emulates the effects of the electrode components of a flat plate capacitive sensor. The distance between the plates due to an applied load is the sensing mechanism. Outside dimensions of 24 × 24 mm and square planar coils with 0.38 mm pitch are formed in the above approach. The load testing results showed good synchronization between the load and capacitance data, except for an 8.3 s delay in the capacitance measurement during unloading, which is most likely caused by material hysteresis.

Ear prostheses are alternatives that allow a patient to recover both the functionality and appearance of a natural ear [120]. PVDF has been used in sensor manufacturing due to its high piezoelectric, pyroelectric, ferroelectric and photopyroelectric properties [121,122,123].

**Figure 4:**
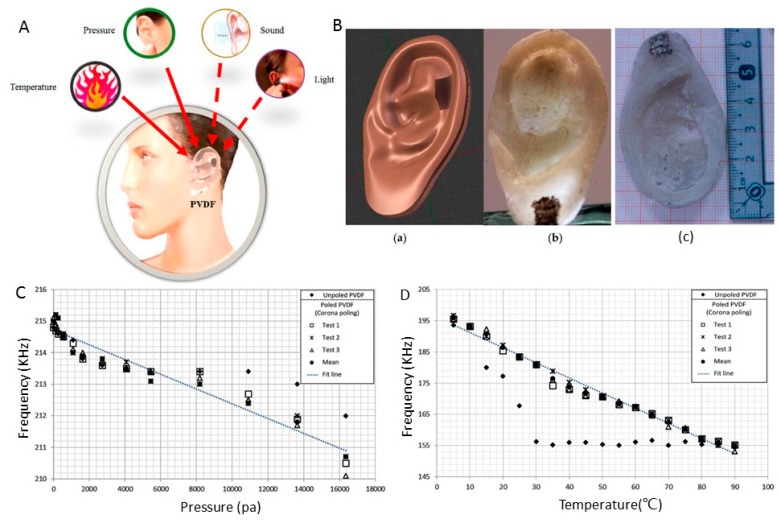
(**A**) Diagram of different stimuli applied to the PVDF prosthesis; (**B**) Human ear created with a 3D CAD program (**a**) and ear prosthesis printed from PVDF (**b**) and (**c**); (**C**) Response of the PVDF prosthesis as a pressure sensor from 0 to 16,350 Pa; (**D**) Thermal response of PVDF prostheses from 2 °C to 90 °C. Reproduced from [13], with permission by the authors © 2016; licensee MDPI, Basel, Switzerland.

Suaste-Gómez and teammates designed an ear prosthesis using 3D computer graphics software and fabricated it using a 3D-printing process with polyvinylidene fluoride (PVDF), as shown in Figure 4B [13]. The prosthesis response to pressure and temperature was observed by detecting the pyroelectric and piezoelectric properties of the PVDF. The results showed that the printed prosthesis is reliable for use under different pressure (0 Pa to 16,350 Pa) (Figure 4C) and temperature (2 °C to 90 °C) conditions (Figure 4D). These excellent studies demonstrated that the 3D-printed ear prosthesis has great potential in the biomedical engineering field.

Fiber Bragg grating (FBG) was used as the pressure-sensing element embedded in a 3D-printed acrylonitrile butadiene styrene (ABS) body, resulting in a new type of pressure sensor [14]. Increasing or decreasing the pressure to simulate different FBG wavelength changes the sensing mechanism. 3D-printing technology has made the process simple, rich in variability and attainable at low cost. Lin and coworkers packaged FBG on a cylindrical pressure sensor for pressure testing and found a linear relationship between the wavelength of the FBG sensor and the pressure loading during a static water pressure experiment at pressure loadings of 0–4 bar. The sensitivity of the cylindrical pressure sensor was nearly 0.208 nm/bar. The results from COMSOL software simulation analysis and experiments were also compared.

#### 3.1.3. Tactile Sensors

Conductive inks have been implemented for a variety of capacitive sensing applications, including strain sensors [7,9,113], humidity sensors [124] and tactile sensors [125,126]. Bulk copper interconnects have the potential to fill the role of these conductive inks due to their high bulk conductivity and elimination of high temperature curing and robust interconnects to microcontrollers or other components. Advancements in the area of thermoplastic 3D printing have facilitated the embedding of bulk wire and mesh into polymer-based 3D-printed structures.

Shemelya and coworkers [15] fabricated tactile sensors by encapsulating copper wire and copper mesh capacitance devices in 3D-printed structures. These devices create the basic framework for the optimized and direct integration of bulk copper capacitive devices into an extrusion-based process. Sensor (1) used a single copper wire 320 μm in diameter for capacitive sensing. Sensor (2) replaced the single wire with copper mesh. Sensor (3) was fabricated using a polycarbonate (PC) polymer material and was implemented as a fully embedded device with an encapsulated copper mesh sensor, zener diode, resistors, LEDs, and electrical connections. Sensor (3) demonstrated the feasibility of an embedded capacitance sensor in complex 3D applications or those requiring hermetic sealing [127]. As the results shown, all of the sensors were easily able to distinguish each material based on relative capacitance, and an increased embedded sensor depth decreased the device sensitivity in a linear fashion [16].

**Figure 5:**
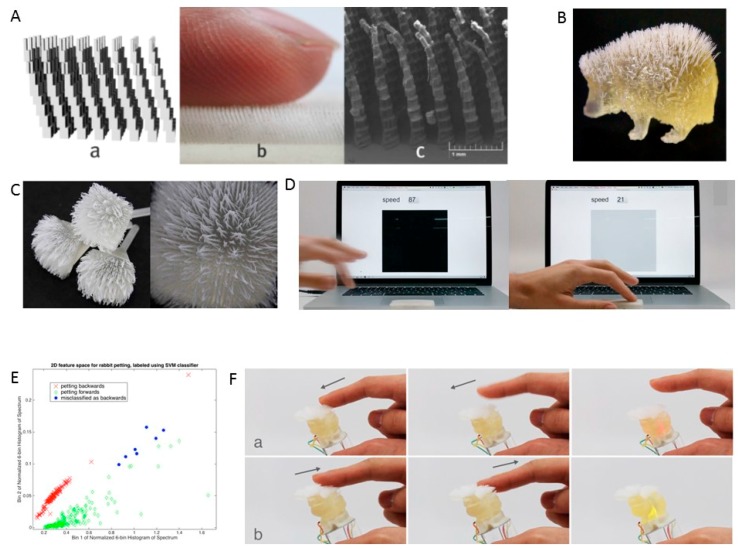
(**A**) Computer visualization (**a**) of printed hair, with close view (**b**) and SEM photo (**c**) of actual printed hair; (**B**) Hedgehog with printed hair; (**C**) Printed hair arrays on curved surfaces; (**D**) Swiping speed mapped to the gray scale of the block; (**E**) 2D feature space for rabbit petting labeled using SVM classifier; (**F**) Direction of swiping on the hairy surface can be differentiated. When one swipes along the hair direction, the bunny turns green; when one swipes against the hair direction, it turns red. Reproduced from [17], with permission from ACM © 2016.

Ou and teammates [17] presented a method for the 3D printing of hair-like structures on both flat and curved surfaces. The designed and fabricated hair geometries are smaller than 100 microns (Figure 5A), and the ability to fabricate customized hair-like structures is shown in Figure 5B,C. The 3D-printed hair can be used in the design of passive tactile and swipe sensors. The researchers tested the real-time pipeline using a simple demo application that estimates and displays the speed in real time using the audio input of the computer to capture the signal (Figure 5D). Forwards and backwards swipes along the back of a model of a rabbit were recorded using a support vector machine (SVM) with a linear kernel (Figure 5E). The ability to sense finger swiping on the hair made this tactile sensor suitable for the design of interactive toys that seamlessly combines a sensing mechanism with surface texture. Jifei Ou fabricated a furry bunny that demonstrates the correct way to pet animals by changing the LED color inside its body (Figure 5F). This method has the potential to classify additional gestures, including multi-finger gestures, location of touch, and intensity of touch and can also be used in the design of everyday interactive objects.

#### 3.1.4. Displacement Sensors

Eddy current sensors are often used in the non-destructive testing of conductive objects. These devices facilitate the examination of surface breakage, subsurface discontinuities [128], fatigue cracks [129], material wear-out [127], and profile imagery [130]. In general, the most common applications of the eddy current sensor are displacement sensors [131]. The detection of the impedance changes in a sensing coil in the presence of a moving conductive target is the sensing mechanism. The maximum measurement distance of a coil-target is almost one half of the coil diameter. Eddy current-based sensors offer the advantages of non-contact, high temperature range, insensitivity to dirt and humidity, and no need for magnetic materials [18].

Jeranče et al. [18] designed and fabricated an eddy current displacement sensor by ink-jet printing of silver ink on a flexible substrate. The oscillating frequency was measured by a microcontroller when the inductor was connected with an oscillator circuit. A simple approximate simulation method was presented, and the displacement of a large target perpendicular to the plane of the inductor was studied at frequencies between 1 and 20 MHz. The sensitivity for position measurement depends on the distance from the target. The number of rising edges detected per 100 ms of the oscillating frequency was measured with respect to the distance between the sensor and aluminum plate. Even a small steel ball 8 mm in diameter can be reliably detected by measuring the distance from the center of the sensing element. This design is highly dependent on the limitations of the conductor printing. With progress in printing technology, this method is likely to be used in many new applications.

**Figure 6:**
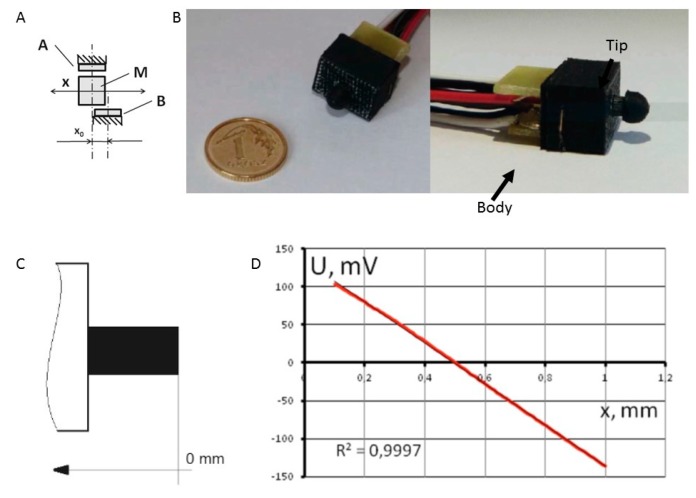
(**A**) Configuration of the differential transducer with Hall-effect sensors—one magnet system applied in the technology demonstrator: M—micromagnet, A and B—sensors, x—linear displacement, x_0_—phase shift at maximum sensitivity of Hall sensors; (**B**) Visualization and (**C**) design of the sensor; (**D**) Physical experiments—experimental characteristics of the sensor. Reproduced from [19], with permission from Springer International Publishing AG, Cham, Switzerland © 2016.

Bodnicki and teammates [19] built miniature converters for the measurement of micro-displacements. These displacement transducers were elaborated and manufactured based on miniature permanent Nd-Fe-B magnets and Hall-effect devices using 3D-printing technology. A configuration of two Hall sensors and one micro-magnet system that generates signals is presented in Figure 6A. All of the components in the sensor were manufactured with 3D-printing techniques, except for a reverse spring made of bronze (Figure 6B). The output signal exhibited good linearity (Figure 6D). Tests of the displacement sensors demonstrated the potential to achieve satisfactory resolution of at least 0.3 micrometer at a displacement range of 1 millimeter. Systems consisting of such sensors are expected to enable the construction of measurement arrays or matrixes of relatively high information density.

#### 3.1.5. Accelerometers

An accelerometer is a device that measures coordinate acceleration, which is the rate of change of velocity. Accelerometers have multiple applications in industry and science. Highly sensitive accelerometers are components of inertial navigation in machinery, aircraft, medical instruments and missiles.

Palma and coworkers [20] presented a manufacturing process for the printing of suspended structures that could be used as capacitive accelerometers. These cantilevers were fabricated via screen-printing on PET with the result of delivering more flexibility to the final sensors. The researchers used a commercial polyvinyl alcohol (PVA) film, which can be removed by water, as the sacrificial substrate. Silver paste used as the structural material showed better performance during the removal process for the sacrificial substrate than the previous approach based on an acetone bath. The peak-to-peak displacement at the free end of the cantilever was measured as a function of acceleration at different frequencies. To test the fabrication process, the same design of cantilever was printed except for changes in the gap between the substrate and the suspended structure from 120 μm to approximately 40 μm. The peak-to-peak displacement of these two types of cantilevers at 10 Hz. The use of biodegradable PVA materials rendered the process more environmental friendly. The cantilever dimensions can be customized by changing the printing parameters.

**Figure 7:**
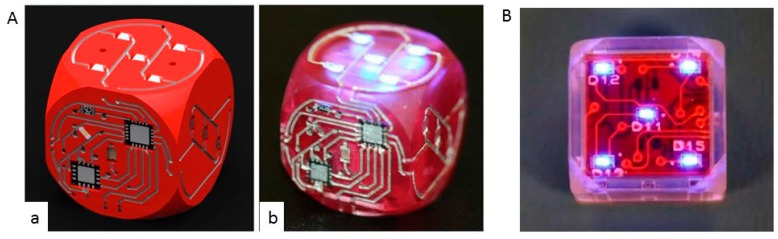
(**A**) CAD and actual fabricated die; (**B**) Final die with a housing fabricated by traditional manufacturing. Reproduced from [21], with permission from © 2014 IEEE.

MacDonald et al. [21] developed a novelty six-sided gaming die that includes a microprocessor and accelerometer. The die can detect motion and upon the cessation of motion can identify the top surface through gravity and illuminate light-emitting diodes (Figure 7A). The development cycle was reduced from weeks to hours by including 3D-printing techniques. A plastic injection molded case was used to manufacture the housing of the die (Figure 7B). This dice has a normal physical dimensions (17 mm or 19 mm per side).

#### 3.1.6. Angular Sensors

**Figure 8:**
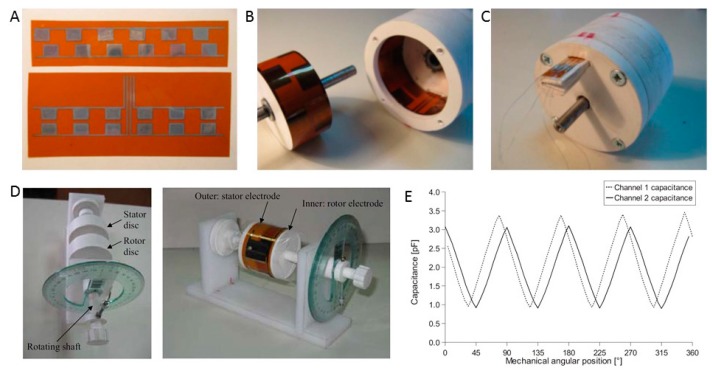
(**A**) Unrolled topology of the stator and rotor silver printed electrodes on flexible foil (Kapton film); (**B**) Picture of the dismantled sensor; (**C**) Picture of the assembled sensor. Reproduced from [22], with permission from Advances in Electrical and Computer Engineering © 2016; (**D**) In-house developed platform (**a**) and mounted foils and final sensor structure (**b**); (**E**) Measured capacitance for the sensor prototype with one full-turn measurement range. Reproduced from [23], with permission from Emerald Group Publishing Limited.

Some inkjet-printed two dimensional (2D) plane sensors can be folded into 3D structures for angular position sensing. Krklješ and coworkers [22] presented a capacitive angular position or velocity sensor that exploits the advantages of flexible and printed electronics. Every sensor has a resolution of six pulses per full turn (Figure 8A). The main component of the angular-speed sensor represents two ink-jet electrodes that were printed on a flexible substrate (Kapton film foil). The substrate is 50 μm thick with a dielectric constant of 3.2. After printing and drying, the substrates with the printed conductive capacitive electrodes were wrapped around the stator and rotor components of the platform (Figure 8B). These sensors belong to the incremental encoder type with two quadrature channels. The sensor’s mechanical construction consists of five components, namely, a stator, a rotor, a shaft, ball bearings and a lid (Figure 8B,C). Figure 8D shows the mounted foils and the final sensor structure [23]. The measured capacitance as a function of the angular position for both channels is depicted in Figure 8E. Nevertheless, certain small disagreements exist that might be a consequence of mechanical inaccuracies in the sensor platform and inaccuracies in the positioning of the Kapton foil. This design offers simple sensor replacement and an uncomplicated mounting procedure.

In a report by Van Tiem [24], a biomimetic angular acceleration sensor was developed. This sensor has a fluid-filled circular channel, and the fluid flows relative to the channel when exposed to angular acceleration. Electromagnetic flow sensing is the sensing mechanism. The two 3D-printed components form a channel that allows for ease of mounting for magnets and electrodes. The electrodes are used to measure the flow-induced potential difference, and experiments demonstrated an acceleration-dependent output voltage. The angular acceleration sensor was mounted to a rotation table for subsequent testing in which the frequency of rotation was set using a function generator. The angular acceleration is exposed to inertia-driven fluid flows, which can be used in rotational acceleration sensing. The measurements were performed with tap water at room temperature when set with a low-pass filter.

### 3.2. Acoustic and Ultrasonic Sensors

**Figure 9:**
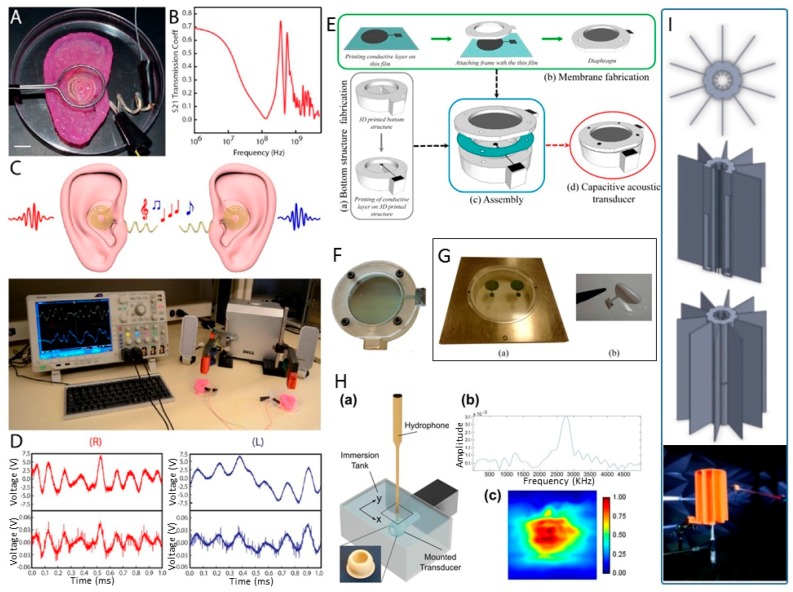
(**A**–**D**) Electrical characterization of the bionic ear. Reproduced from [25], with permission from the American Chemical Society © 2013; (**E**) Fabrication steps of acoustic sensor combining 2D inkjet printing and 3D printing techniques; (**F**) Printed capacitive acoustic transducer; (**G**) Inkjet printing on thin Mylar film. Reproduced from [26], with permission from by the authors © 2015; licensee MDPI, Basel, Switzerland; (**H**) The hollow, spherical ceramic shell performing as an ultrasonic transducer. Reproduced from [27], with permission from the authors © 2015, Phys. Status Solidi A, published by WILEY–VCH Verlag GmbH & Co. KGaA, Weinheim, Germany; (**I**) Designed Near-field monaural localization structure. Reproduced from [28], with permission from by the authors © 2015; licensee MDPI, Basel, Switzerland.

Mannoor and teammates [25] presented a novel strategy for the additive manufacturing of biological cells within structures and nanoparticle-derived electronic elements and generated a bionic ear through the 3D printing of a cell-seeded hydrogel matrix using a geometric imitation of a human ear (Figure 9A). Silver nanoparticles were infused during the printing process. This method allows the in vitro culture of cartilage tissue in the printed ear. Cochlea-shaped electrodes were assembled with the ear to enable the readout of inductively coupled signals. The S21 (forward transmission coefficient) parameter of the coil antenna was analyzed and found to transmit signals across an extended frequency spectrum (Figure 9B). Magnetic loop antennas with ferrite cores were applied to transmit the left and right channels of stereophonic audio to the left and right bionic ears (Figure 9C,D). The structure of the printed ear exhibited enhanced auditory sensing for radio frequency reception. This strategy represented the principle of intertwining the multi-functionality of additive manufacturing techniques with nanoparticles and tissue engineering concepts.

Boddaert and coworkers [26] presented a new approach that combined 3D-printing and direct-write 2D inkjet printing techniques to fabricate capacitive acoustic transducers. The bottom structure, with a rigid backplate of small radius and a cavity, was fabricated using 3D-printing technology, and a silver layer was produced on the 3D-printed structure via the ink-jet printing technique. The complete fabrication steps are illustrated in Figure 9E, and a prototype of the printed capacitive acoustic sensor is presented in Figure 9F. Figure 9G shows a specific film fabricated as a pre-stressed membrane via inkjet printing. Measurements showed that the printed acoustic transducer operates as a capacitor and produces high sensitivity and selectivity at its first resonance frequency. Testing conducted on the capacitive acoustic sensor with a membrane radius of 8.1 mm, a backplate radius of 871 μm, cavity height of 3990 μm, an air gap of 67.7 μm, membrane thickness of 23 μm, membrane tension of 48 N/m, a Q-factor of 34 at its frequency of 3490 Hz, and a static capacitance of 0.76 pF showed satisfactory proximity to the numerical results.

An ultrasonic transducer was fabricated by Woodward’s group using a low-cost micro-stereolithography (SLA) technique [27]. The chosen light-sensitive material contained Pb_0.65_ (Mg_1/3_Nb_2/3_) O_3–0.35_PbTiO_3_(PMNT), known as a material with one of the highest piezoelectric coefficients, and a light-sensitive polymer. A hollow spherical shell structure of the piezoelectric material was produced without tooling or additional equipment and was capable of generating ultrasound in the MHz range (Figure 9H).

A near-field sound localization device fabricated based on the small profile monaural structure was reported by Kim’s group [28]. The near-field monaural localization (NFML) structure with devised lengths for the pipes and center body is presented in Figure 9I. The NFML structure around the microphones produces direction-wise spectral variation, and the sensing mechanism identifies and estimates the position of the sound source. The dimensions of the structure are 14 cm for the wing-to-wing diameter, 5 cm for the main body diameter, and 19 cm for the depth. These dimensions are significantly reduced compared with those in previous studies [132].

### 3.3. Optical Sensors

**Figure 10:**
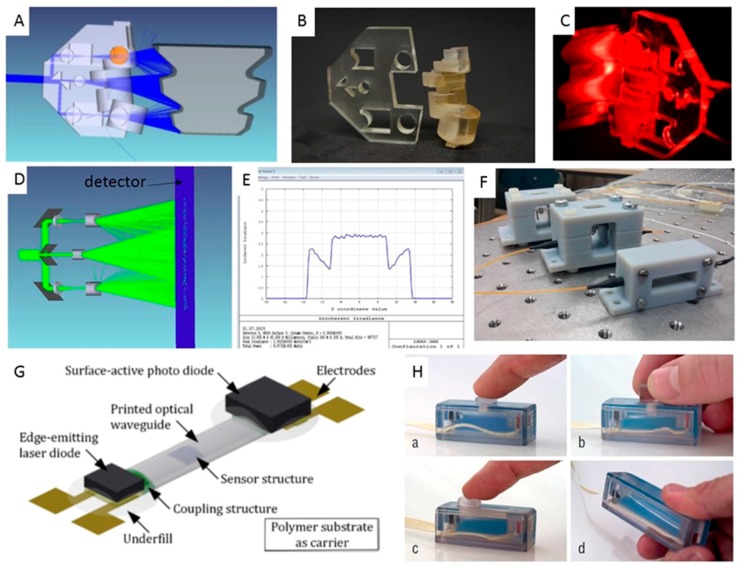
(**A**) Simulation of the freeform optical sensor illuminating the sample; (**B**) Photograph of freeform sensor and cylindrical lens array; (**C**) Freeform optical set up illuminating a metal sample. (**D**,**E**) Representation of the splitting and diverging process of freeform optical sensor. Reproduced from [29], with permission from SPIE © 2015; (**F**) Photograph of the fiber optic vibration sensors. Reproduced from [30], with permission from SPIE © 2016; (**G**) Drawing of planar optronic sensor system. Reproduced from [31], with permission from Elsevier Ltd. © 2015; (**H**) User inputs such as push (**a**), rotation(**b**), linear movement (**c**), and acceleration (**d**) can be sensed by the displacement of a 3D printed light guide. Reproduced from [32], with permission from ACM © 2012.

3D-printed free-form optical sensors are a different and new approach for optical metrology [29]. All optical elements, such as mirrors and lenses, can be reduced to one simple printed sample with the ability to illuminate a complex-shaped component for shape measurement. Maillard and Heinrich [29] printed free-form optical sensors for a metrology application by designing a free-form optical sensor combined with a cylindrical array that can be used to observe a continuous laser line over the surface of the sample (Figure 10A) with no shadowing effects. The design was fabricated using a 3D printer, as shown in Figure 10B. In the experiment, the sensor generated a laser line over the component surface (Figure 10C), and the ray trace analysis of the freeform sensor is illustrated in Figure 10D. The results show a flat-hat profile in the central zone and an increasing profile at the sides of the flat-hat energy distribution (Figure 10E).

Igrec and colleagues [30] demonstrated a lightweight and inexpensive fiber-optic vibration sensor for high-power electric machines using 3D-printing technology (Figure 10F). Modulation of the light intensity using a blade attached to a bendable membrane is the sensing mechanism used in this application. The sensor shows the best performance of linear response at low bandwidth (<150 Hz). The fiber-optic vibration sensors developed in this method are simple to assemble, adjust, calibrate and repair.

Printing processes are applied in the creation of three-dimensional transparent structures that are used as multimode optical waveguides in intelligent systems. The end facets of waveguides serve as interfaces to adjacent functional elements [31].

Wolfer et al. [31] investigated and demonstrated that 3D-printing technology is suitable for creating polymer optical waveguides as the central elements of planar optronic systems (Figure 10G). The methods reported form a process chain that combines high-throughput flexographic techniques and inkjet printing technology for the efficient mass production of customized elements for individualized sensor systems [31]. The Willis group presented an approach to the fabrication of optical elements for interactive devices via 3D printing [32] in which optics are embedded in the casing or mechanical structure for sensing, display, and illumination (Figure 10H).

### 3.4. Electromagnetic Sensors

Frequency selective surfaces (FSS) are generally described as two-dimensional structures that filter electromagnetic waves over a range of frequencies. Certain volumetric or three-dimensional frequency selective structures offer superior performance over the traditional FSS [133,134].

**Figure 11:**
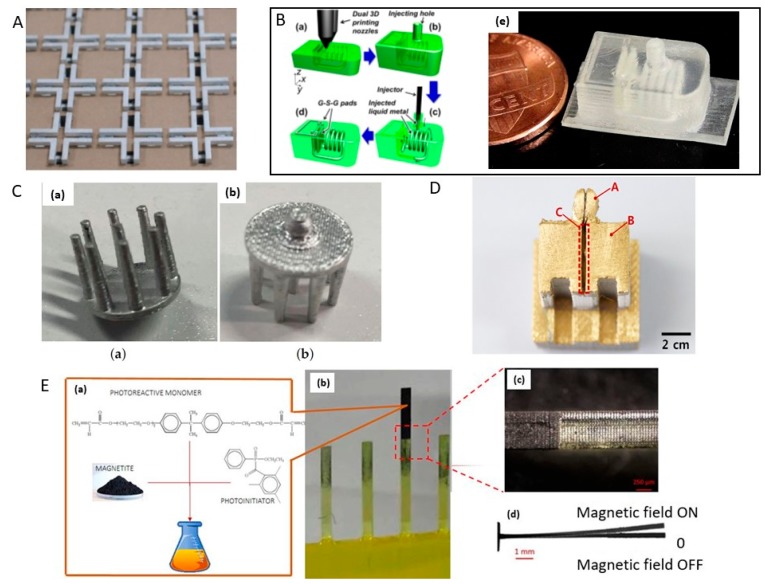
(**A**) A 3 × 3 array of the 3D folded loop FSS. Reproduced from [33], with permission from IEEE © 2013; (**B**) 3D printing to form structures with hollow channels and chambers (**a**). A finished 3D structure with the injection hole (**b**). Liquid metal filling (**c**), and surface planarization to remove the injection hole and extra metal (**d**). Optical photos of the 3D printed structures (**e**). Reproduced from [34], with permission from IEEE © 2015; (**C**) Example of a 3D printed EEG electrode coated with silver paint. Reproduced from [35], with permission from the authors © 2016; licensee MDPI, Basel, Switzerland; (**D**) A picture of 3D printed object deposited with Ti/Au. Reproduced from [36], with permission from IEEE © 2015; (**E**) Array of cantilevers (**b**); The high precision at the interface of the two resins (**c**); Example of superimposition of optical images acquired for the sample in its initial position (0) and when the magnet approached (**d**). Reproduced from [38], with permission from the American Chemical Society © 2016.

Sanz-Izquierdo and Parker [33] fabricated a novel frequency selective electromagnetic structure in parallel and integrated it using the additive manufacture of buildings for the control of electromagnetic wave propagation. The cores of these structures were fabricated using a 3D printer with a plaster-based material that establishes the specific electromagnetic architecture of a specific building (Figure 11A). The theoretical and experimental results all confirmed the operation of surfaces within the UHF frequency band. Using this method, larger array sizes could be fabricated using a robotic automatic system for additional applications.

Wu and teammates [34] presented wireless inductor-capacitor (LC) tank sensors constructed using 3D printing. The fabrication process is shown in Figure 11B in which hollow channels in the 3D structure are filled with liquid metal (silver particles) to form inductors and capacitors. A radiofrequency (RF) LC tank with a resonance frequency of 0.53 GHz was realized by this method.

Kalyanasundaram and Arunachalam [135] developed a miniature conformal sensor for real-time electric field measurement. The sensor was screen-printed on a flexible thin substrate. The flexible screen-printed miniature sensors have a 0.9 MΩ/m line resistance, 1 mm line width and 1.5 mm line spacing. Characterizations of the sensor show good agreement with the design equations and 3D numerical simulations, meaning that the fabrication process is practical. The experimental results indicated that an array of miniature conformal electric field sensors could be effectively used in in situ monitoring in microwave nondestructive testing.

#### 3.4.1. EEG Sensors

Electroencephalography (EEG) is be used to record brain activity in a non-invasive manner. In recent years, wearable EEGs have been used in out-of-the-lab applications for epilepsy diagnosis, stroke rehabilitation, and brain-machine Interfaces. Wetting is a significant obstacle for these EEGs, and invasive electrodes have the potential for skin irritation and poor long-term stability. Krachunov and Casson [35] developed dry electrodes used in electroencephalography (Figure 11C) by fabricating the sensors using a low-cost desktop 3D printer with commercial components, allowing for quick and inexpensive electrode manufacturing. The test results showed that the performance of these 3D-printed EEG electrodes is suitable for BCI applications despite the existence of noise.

Cho group [36] designed a non-invasive multichannel EEG recording system that implements the 3D-printing technique to record signals from underwater animals such as zebrafish (Figure 11D). The fabrication of this sensor is cost and time efficient. Figure 11D shows the printed object created using metal deposition of Ti/Au without damage during the process.

#### 3.4.2. Magnetic Field Sensors

Polzinger and teammates [37] fabricated magnetic field sensors based on toroidal core coils which printed by Aerosol Jet^®^ techniques. Nano silver and cooper ink were used for printing the coil winding. Final sensors of toroidal core coils with Aerosol Jet^®^ printed windings and dispensed ferrite core on PCB substrates. In their experiments, up to 39 winding numbers can be achieved. The line width of the coil windings is 70 μm, and the layer thickness is 5–10 μm. The electric resistances of the coils can be controlled between 2 and 20 Ω.

Caterina Credi and colleagues [38] provide a method to fabricate cantilever-type microstructures by stereolithography of ferromagnetic photopolymers used as possible magnetic field sensors (Figure 11E). The sensing performance in terms of static deflection vs applied magnetic field is the sensing mechanism, and was qualitatively studied in theirs research. The polymeric cantilevers-shaped microstructures with high aspect ratio were obtained by the layer-by-layer fabrication and the special self-standing characteristic of the resins (Figure 11E(b)). The high precision at the interface of the two resins achieved micrometric control of the printing process (Figure 11E(c)). These methods avoid expensive and time-consuming fabrication processes. The highest resolution of printed micro-magnetic cantilever-based structures was obtained for samples with a length of 9 mm and rectangular cross sections of 0.6 mm× 0.2 mm (width ×thickness). The combination of SL-printing with the formulation of novel smart ferromagnetic photopolymers open the new way for fabricating high-customized complex 3D magnetic sensors.

#### 3.4.3. Antennas

Antennas act as effective transducers between free space and guided waves over a range of frequencies due to their impedance bandwidth [39]. However, most small antennas operate as a single resistor-inductor-capacitor (RLC) circuit. The bandwidth of these antennas is inversely proportional to their radiation quality factor (Q), which is defined as the ratio of energy stored to energy radiated [136]. A fundamental relationship exists between the antenna size and Q [137,138]. The bounding limit of the antenna performance depends on the electrical size of the antenna [139].

Nassar and team [140] designed a wide-band harmonic transceiver using 3D-printed technology for embedded passive wireless monitoring. The wires are printed vertically on a cambered surface, and the substrate is fabricated using fused deposition modeling. By integrating other devices on the substrate, the device features a wide band and facilitates harmonic transceiver micro-integration. Compared with a design fabricated using printed circuit board (PCB) technology, the 3 dB conversion efficiency bandwidth is increased from 2.5 to 19.2%, the weight is reduced by 40% and the conversion efficiency at an RF input power of −20 dBm is reduced by 5.5 dB [40].

**Figure 12:**
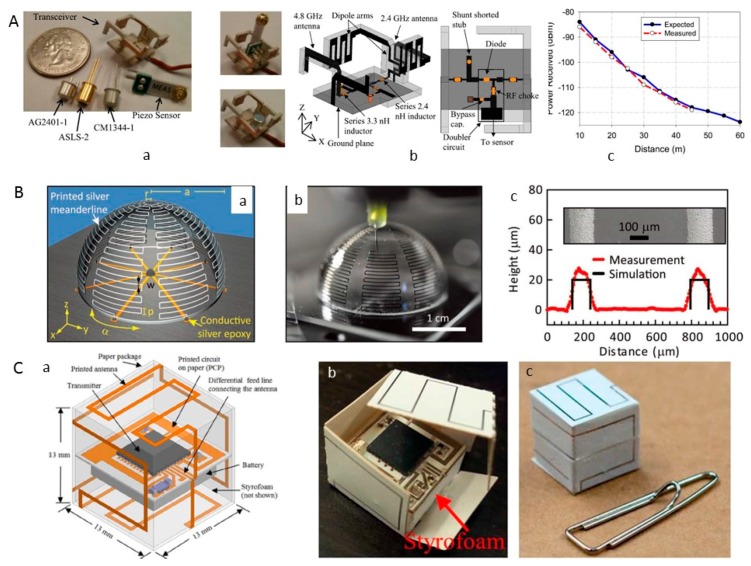
(**A**) Photograph of the fabricated transceiver and the sensors (**a**), a CAD illustration of the transceiver design (**b**), the measured and expected received return signal strength versus distance using a 43 dBm EIRP transmitter and 11 dBi gain receiver (**c**). Reproduced from [41], with permission from IEEE © 2015; (**B**) Schematic illustration of an electrically small antenna with labeled geometric parameters (**a**) and the optical image of an antenna during the printing process (**b**), optical profilometry scan of representative meanderlines on electrically small antennas with the background surface subtracted and scanning electron microscopy image of these features (inset). Reproduced from [39], with permission from WILEY-VCH Verlag GmbH & Co. KGaA, Weinheim, Germany, © 2011; (**C**) Sensor model (**a**) and completed sensor (**b**,**c**). Reproduced from [43], with permission from IEEE © 2014.

Nassar and co-workers [41] presented a compact, energy-efficient, passive, and wireless vibration sensor node for embedded monitoring (Figure 12A). By combining 3D machined substrate small antennas on the transceiver, a device was fabricated within a sphere with a diameter of 21 mm (Figure 12A(a)). The system is equipped with a frequency doubler on the sensor node to return the second harmonic of the interrogation signal (Figure 12A(b)). To deliver and collect the maximum power to/from the diodes, the antennas are designed to be conjugate matched with the doubler input/output impedances. The sensor node operates by receiving an interrogation signal at 2.4 GHz, doubling the frequency, and transmitting back an amplitude-modulated signal at 4.8 GHz and is optimized for radio frequency input power between −30 and −20 dBm (Figure 12A(b)). The measured efficiency of the node (λ/6 at 2.4 GHz) is 10% at −20 dBm input power. Test results show that vibrations of <0.1 Hz frequency and 0.01 g amplitude can be detected. Using a 43 dBm effective isotropic radiated power transmitter, the communication range is greater than 60 m (Figure 12A(c)), which revealed that the proposed vibration sensor nodes are good candidates for long-range embedded passive sensing.

Adams fabricated electrically small antennas using the conformal printing of metallic inks onto convex and concave hemispherical surfaces in the form of conductive meander lines (Figure 12B). When interconnected with a feed line and ground plane, the resulting 3D ESAs exhibit performance properties that nearly match those predicted theoretically for these optimized designs [39].

Garcia Lopez and coworkers [42] fabricated a small volcano smoke antenna optimized using 3D-printing technology in steel at a low cost. The simulation and measurement of the reflection coefficient show that 3D printing can be used as a method for the fabrication of complex-shaped antennas.

Farooqui and group [43] used inkjet printing technology to print lightweight 3D Lagrangian sensors to address the real-time monitoring of flooding. The antenna is inkjet printed on Kodak photo paper using UTDAg silver nano-ink with UTDots made from silver nanoparticles with an average size of 10 nm dispersed in an organic solvent (Figure 12C). The same procedure is used to print the transmitter circuit on paper. The antenna performance was demonstrated in an unconventional lossy medium of water, and the sensor was able to communicate across a decent range both in air and half immersed in water.

### 3.5. Bio- and Chemical-Detection Sensors

#### 3.5.1. Biosensors

Biosensors are small devices that use biochemical molecular recognition properties as the basis for selective analysis [141]. The greatest difference between biosensors and other sensors is that the signal detection of biosensors contains sensitive substances. In recent decades, we have witnessed a tremendous number of activities in the area of biosensors. Due to characteristics such as intelligence, miniaturization, and specificity, biosensors offer exciting opportunities for researchers and corporations in applications from situ analysis to home self-testing. The biomaterial patterning of biosensor fabrication is one of the most promising techniques for improving biosensor stability. 3D-printing technology is reliable and efficient for facilitating controllability over the entire process and represents an authentic breakthrough for the development and mass production of biosensors. Mandon and teammates [44] presented a study that demonstrated the capacity of one 3D-printing technique, i.e., digital light processing (DLP), to produce hydrogel sensing layers with 3D shapes that were unattainable using conventional molding procedures. The sensing layer model was composed of a sequential enzymatic reaction that generated a chemiluminescent signal in the presence of glucose and luminol. This research represents a path to a completely new sphere of development of multiplex sensing layers that are printed separately and assembled on demand to create complex sensing systems.

**Figure 13:**
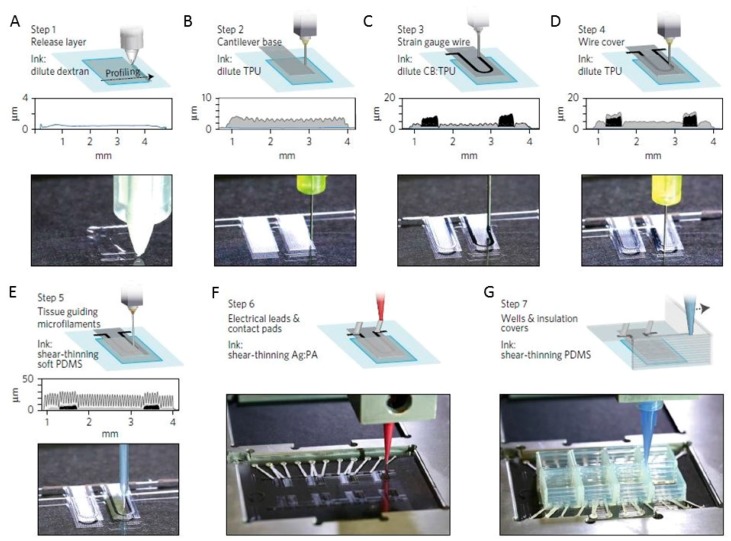
Device principle and microscale 3D-printing procedure. (**A**) In print step 1, a 0.5-μm dextran thin-film sacrificial layer is printed; (**B**) In print step 2, a 3 μm TPU thin-film cantilever base is printed; (**C**) In print step 3, a 6.5-μm-thick CB:TPU strain sensor loop is added to the cantilever base; (**D**) In print step 4, a 1.5-μm TPU wire cover is added; (**E**) In print step 5, 20-μm-tall, 60-μm-wide PDMS microfilaments are printed in slightly overlapping lines. The filaments constitute the top part of the cantilever and guide cardiomyocytes to form anisotropic laminar tissues; (**F**) In print step 6, electrical leads and contact are added using a high-conductivity Ag:PA ink; (**G**) In print step 7, covers to insulate exposed wires and wells to contain cells and media are printed using PDMS, PLA or ABS. Reproduced from [45], with permission from © Macmillan Publishers Limited, part of Springer Nature.

Recently, microphysiological systems (MPS), also known as organs-on-chips, that recapitulate the structure and function of native tissues in vitro, have emerged as a promising alternative [142]. Lind et al. [45] designed and produced an instrumented cardiac microphysiological device via multi-material 3D printing. Six functional inks were fabricated based on piezo-resistive, high-conductance, and biocompatible soft materials that enable the integration of soft strain gauge sensors within micro-architectures that guide the self-assembly of physio-mimetic laminar cardiac tissues (Figure 13).

These printed sensors are non-invasive and can read out the tissue contractile stresses. Drug response can also be monitored using this device. Human stem cell-derived laminar cardiac tissues cultured inside the cell incubator for contractile testing were maintained for four weeks. This research illustrated a programmable microfabrication approach as an alternative method for in vitro tissue engineering, toxicology, and drug screening research. Future biosensor applications will require high performance, including the real-time monitoring of physiological events and will tend to be fully integrated wearable sensor arrays for multiplexed in situ analysis [143,144].

#### 3.5.2. Chemosensors

**Figure 14:**
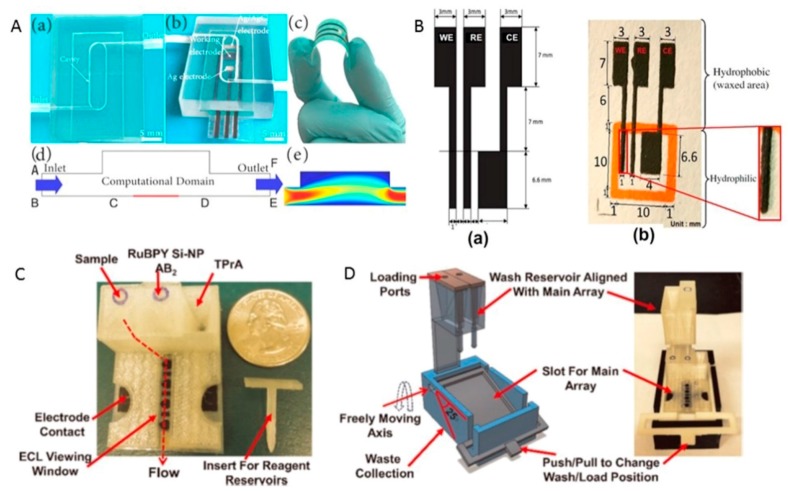
(**A**) Optical images of the devices (**a**,**b**), photograph depicting SPE which is flexible and can be bended easily (**c**), computational domain of microfluidic cell with the work electrode (**d**), profile of microfluidic cell from the side view (**e**). Reproduced from [46], with permission from the American Chemical Society © 2016; (**B**) Layout of three electrodes: (**a**) designed device, (**b**) fabricated device. Reproduced from [47], with permission from Elsevier B.V. © 2012. (**C**,**D**) 3D-printed main array and wash reservoir module. Reproduced from [49], with permission from Elsevier B.V. © 2015.

Hong et al. [46] reported a microfluidic electrochemical sensor that presented superior electrochemical detection properties toward heavy metal ions and could deliver real-time stripping analysis of heavy metal ions. The desired shape of the model for velocity profiles in microfluidic cells was built and optimized using the finite element method (FEM). The electrode of the microfluidic cell was a flexible screen-printed electrode (SPE) (Figure 14A). This novel sensing mechanism solved the problems of SPE-based sensors such as sensitivity, stability and reproducibility.

A new paper-based electrochemical sensor (Figure 14B) was fabricated using an inkjet-printed PANI modified SPCE electrode developed as a low-cost and disposable point-of-care device for pre-screening purposes [47]. The use of SLS has been demonstrated to fabricate electrochemical electrodes that can be used as platform for different electrochemical applications [48].

Kadimisetty and teammates [49] developed a 3D-printed, low-cost, sensitive, and super capacitor-powered electrochemiluminescent (ECL) protein immunoarray. This microfluidic immunoarray (Figure 14C,D) was printed using a commercial desktop 3D fused deposition modeling printer. The immunosensor detects three cancer biomarker proteins in serum within 35 min. The detection limits were 300–500 fg∙mL^−1^ for the three proteins in undiluted calf serum.

This system has a drawback in that a large number of sequential tasks must be completed by the operator to complete an immunoassay. The good performance of this sensor demonstrated that the device is suitable for clinical environments. This work suggests that 3D printing technology can be used to develop more sophisticated immunoarray devices with a higher level of automation. The boundaries among chemical sensors, biosensors, and medical sensors are expected to become increasingly blurred because of more advanced sensors and increasing integration.

#### 3.5.3. Sensors for Food-Quality Monitoring

**Figure 15:**
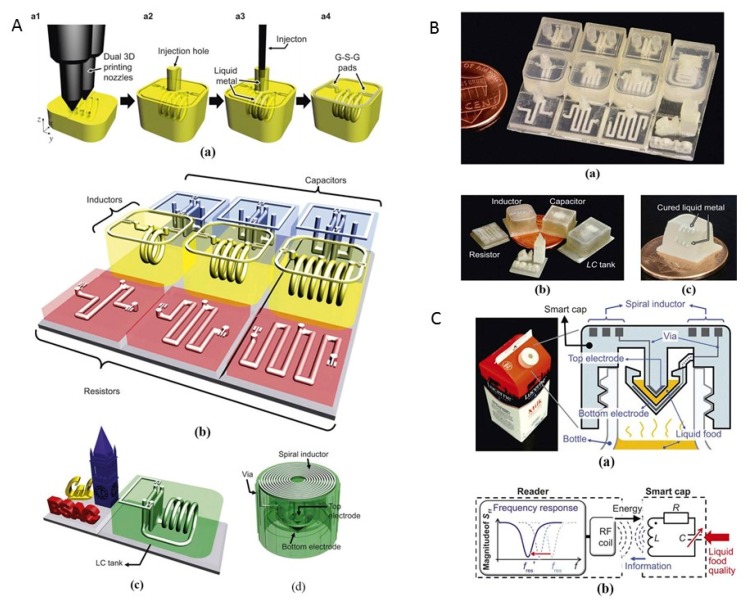
(**A**) Schematic diagram of the additive 3D manufacturing process. The 3D fabrication process with embedded and electrically conductive structures (**a**). 3D microelectronics components, including parallel-plate capacitors, solenoid-type inductors, and meandering-shape resistors (**b**). A 3D LC tank, which is formed by combining a solenoid-type inductor and a parallel-plate capacitor (**c**). A wireless passive sensor demonstration of a “smart cap,” containing the 3D-printed LC-resonant circuit (**d**); (**B**) An optical image of fabricated microelectronics components; (**C**) The proposed “smart cap” for rapid detection of liquid food quality featuring wireless readout. Reproduced from [50], with permission from Macmillan Publishers Limited, part of Springer Nature © 2017.

Wu’s group [50] used a multiple-nozzle printing system with a resolution of 30 μm and combined liquid metal paste filling to produce resistors, capacitors, and inductors. These researchers built many complicated circuitries to form functional systems, including a 3D radio-frequency (RF) passive circuit with an embedded and wirelessly readable inductor that could enable rapid and built-in sensing for food safety detection. The fabrication steps are shown in Figure 15A. Functional 3D structures were designed and constructed using the 3D-printing technique. The hollow micro-channels and cavities were designed in the 3D structures and filled later with liquid metal paste. A hollow solenoid-shaped channel was formed and to facilitate the liquid metal paste-filling step, injection holes were designed as inlet/outlet ports for the solenoid channels. The solenoid inductor has designated cavities for the ground-signal ground (G-S-G) pads on the top surface, which enables direct frequency characterizations of the RLC circuitry. Liquid metal paste was injected into the printed object to form conductive electrical structures after the printing process. The components are illustrated in Figure 15B. Combining these components, a 3D “smart cap” can be fabricated to monitor food quality (milk, juice, etc.).

If the food package is flipped upside down after mounting the “smart cap” on the liquid food packages, the liquid flows into the capacitor gap and acts as a dielectric material. The changes in the dielectric constant lead to a shift in the resonance frequency of the embedded 3D LC circuit used as the sensing mechanism. The resonance frequency can be detected wirelessly using an inductively coupled reader in real time. The experimental results showed a 4.3% frequency shift for a milk package stored at a room temperature environment for 36 h (Figure 15C). This work demonstrated an innovative method for the fabrication of customized 3D systems with embedded electrical structures for various applications.

### 3.6. Gas Detection Sensors

**Figure 16:**
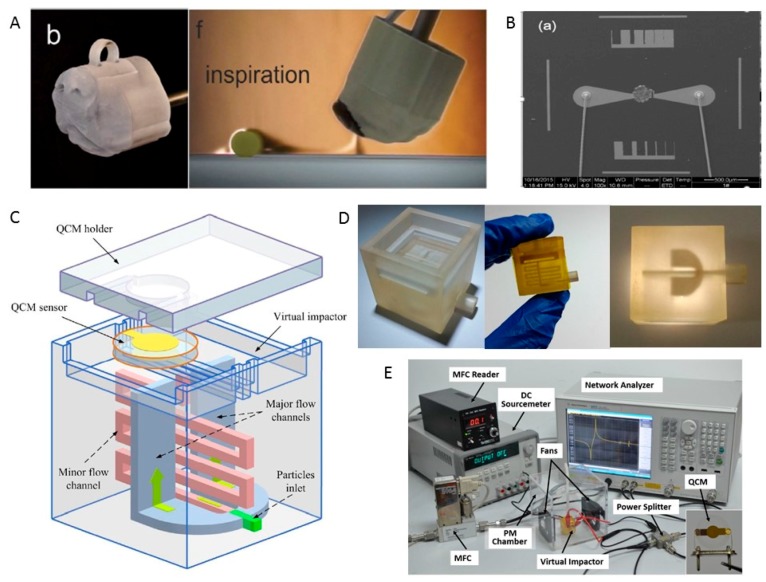
(**A**) 3D printed dog’s nose including a removable PUF insert within the flow path in the vestibule of the nose that collects inspired DNT vapor (**a**) and schlieren image of the 3D printed dog’s nose during the inspiratory phase of sniffing (**b**); Reproduced from [51], with permission from ©2017 Macmillan Publishers Limited, part of Springer Nature (**B**) SEM images of the fabricated sensor with alumina supported bi-metal catalyst deposited on the electrode by screen printing and inkjet printing. Reproduced from [52], with permission from © 2016 Elsevier B.V; (**C**) Schematic of the 3D printed virtual impactor integrated with QCM sensor for detecting airborne particles; (**D**) The virtual impactor fabricated by 3D printed technology; (**E**) Photograph of the experimental setup for prototype characterization. Reproduced from [53], with permission from © 2016 Elsevier B.V.

Staymates and team [51] printed an artificial dog nose using 3D-printing technology (Figure 16A) and combined the artificial nose with fluid visualization experiments to simulate the gas flow of dog breathing. When the dog sniffs, a portion of the gas is pushed into the nasal cavity by inspiration, which extends the “aerodynamic reach”. The connection of the artifact with a commercial explosives detector can increase the detection by a factor of 16.

Lu and group [52] created a quartz MEMS catalytic methane sensor with a back-etched cone cavity using high-resolution abrasive sand blasting together with lift off, screen printing, and inkjet printing (Figure 16B). The microfabrication processes are simplified compared with those of other silicon-based gas sensors. Due to a lower thermal mass beneath the heating and sensing electrode, the sensor performance was enhanced significantly, and the sensor also showed good mechanical stability, including compatibility with contact loading methods of the catalyst and performance against external shock at high temperature. This fabrication process could be applied in harsh environments. Zhao and group [53] created a miniaturization system that can detect tiny particles in air by applying 3D-printing technology and aerodynamic simulation (Figure 16C–E). The impactor fabricated using the 3D-printing process avoids assembly tolerance and maintains accurate alignment. The resonant frequency was shifted when silicon dioxide particles were loaded on the adhesive surface, illustrating the sensing mechanism. After classification, the particles in the major flow area mostly have diameters smaller than 2.5 μm, which verifies the good performance of the virtual impactor. This miniaturized system was demonstrated for use as a low-cost and real-time environmental monitoring tool. This system can perform repeated measurements by removing particles absorbed on the electrode surface of the QCM. Thus, it is possible to realize smaller and more portable PM monitoring according to this approach.

### 3.7. Flow Sensors

**Figure 17:**
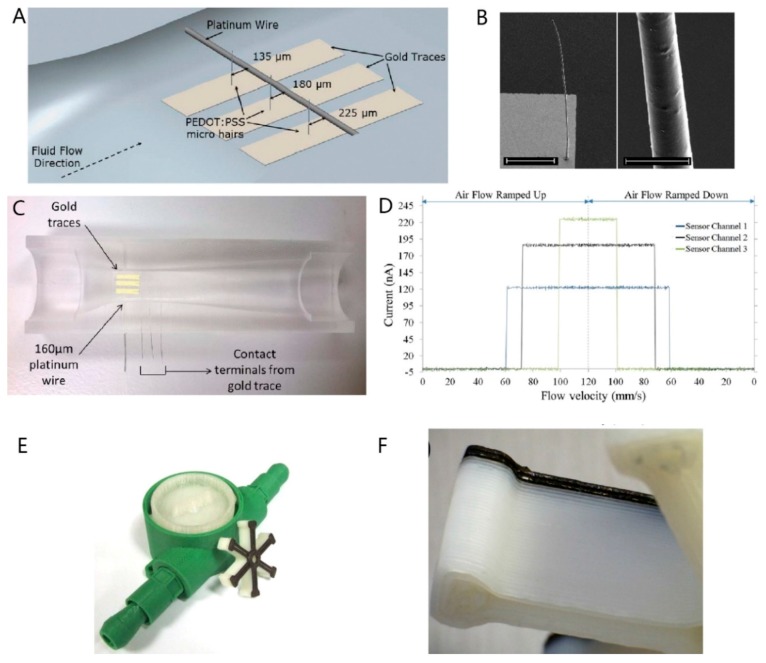
(**A**) Sensor architecture showing the primary components of the micro-hair sensor; (**B**) (**left**) SEM of 1000 μm long micro-hair structure at 45° tilt (scale bar 200 μm) and (**right**) surface of the PEDOT: PSS micro-hair (scale bar 10 μm); (**C**) Cross section of PDMS venturi with gold traces on the disposable sensor substrate; (**D**) Output from 3-channel sensor filtered and shown over a single cycle (ramp up and down) of sensor operation. Reproduced from [54], with permission from © 2015 IEEE; (**E**,**F**) Photograph of the printed flow sensor and impeller. Reproduced from [55], with permission from © 2014 IOP Publishing Ltd.

Flow sensing is an essential technique required for various application environments. Devaraj et al. [54] reported a novel method for the sensing of low-velocity air flow using high aspect-ratio 3D-printed micro-hair structures made of a conducting polymer. The 3D micro-hair structures were printed using a custom-built 3D printer. These high aspect ratio structures offer a larger frontal area to maximize the drag force in an air flow field. In the sensing mechanism, multiple micro-hair structures used as micro-switches respond to air flows (Figure 17A).As represented in Figure 17A, the micro-hairs are printed at an offset of 45 μm from the common contact terminal (platinum wire with 160 μm diameter) of the adjacent micro-hair. The micro-hairs are 1000 μm long and 5.5 ± 0.5 μm in diameter, and SEM images of a single hair are shown in Figure 17B.For the fabrication of a disposable sensor, the sensor substrate is made from a removable 5 mm × 5 mm × 1 mm piece of PDMS with gold traces (Figure 17C). A stereolithographed mold was used to cast the PDMS venturi duct. The connection between the sensor substrate and the common terminal passes through the PDMS cast, and the sensor substrate is placed in a Venturi recess, as shown in Figure 17C. The external data acquisition system can monitor the sensor output continually. The micro-hair structures were subsequently printed on the sensor’s substrate gold traces. Sensor testing results showed that the first micro-hair (located 135 μm away from the common terminal) established contact at 61.52 mm/s, and the second and third micro-hairs made contact at 73.06 mm/s and 99.86 mm/s, respectively (Figure 17D). The estimation of the fatigue point of the sensing element demonstrated that prior to reduced flow response and permanent deformation, these micro-hairs produced a repeatable output over 11,000 cycles. These micro-hairs can also act as micro-switches to create a digital on/off output depending on the air flow velocity. Leigh and colleagues [55] printed a flow-sensor using a magnetite nanoparticle-loaded thermoplastic composite to mimic the function of a commercially available flow-sensing device (Figure 17E,F). This flow-sensor was fabricated using a multi-material 3D printer. The device exhibited a much more linear and predictably accurate response to an increasing flow rate of water. The testing analysis showed no obvious separation of the magnetite composite layers from the underlying ABS layers of the impeller, indicating the robustness of the 3D-printed products. The test results also demonstrated that the printed device has the potential to perform as a conventionally produced sensor.

### 3.8. Temperature- and Humidity-Monitoring Sensors

The sensing of temperature distributions over an area is of interest for applications ranging from medical research [145] and environmental awareness in advanced robots to thermal management systems in satellites. Fast and accurate measurement of the local temperature and humidity with highly spatial resolution is desirable. Conductive inks with 3D-printing technology have been implemented for a variety of capacitive sensing applications that include humidity sensors [56,57,146] and temperature sensors [58,59,60]. A 3D-printed ear prosthesis used as a temperature sensor was mentioned earlier in Figure 4A.

Printed UHF radio frequency identification (RFID) sensor solution was exposed to a certain degree of humidity [56], and typical applications of this printed sensor include noninvasive methods for detecting wetness or humidity levels.

**Figure 18:**
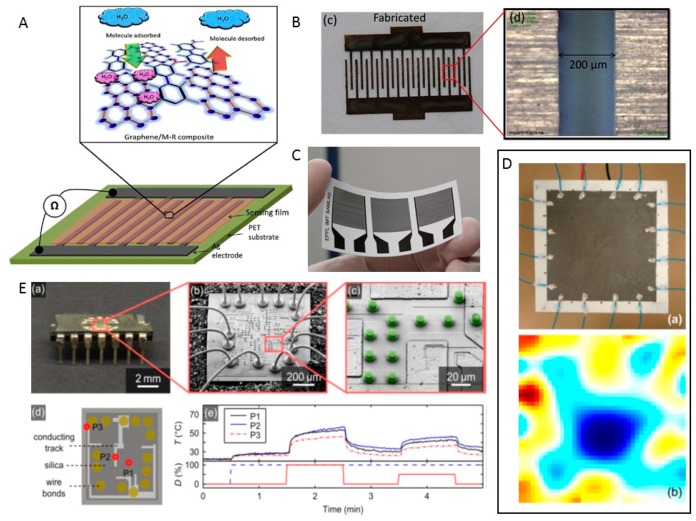
(**A**) Schematic representation of a sensing phenomenon of the proposed humidity Sensor; (**B**) The inkjet-printed sensor’s electrodes with sliver ink. Reproduced from [57], with permission from © 2016 Elsevier Ltd; (**C**) Optical picture of the inkjet-printed capacitors and resistor on paper. Reproduced from [58], with permission from © 2011 IEEE; (**D**) Overhead view of the continuous sensor (black) on a carbon substrate (**a**), and thermal image produced by EIT formed upon cooling the center of the continuous sensor from 68.5 °C (red) to 7 °C (blue) using a Peltier element. Reproduced from [59], with permission from © 2015 WILEY-VCH Verlag GmbH & Co. KGaA, Weinheim; (**E**) Photograph of an IC 7400N prepared for DLW (**a**), Scanning electron micrograph (SEM) of temperature probes on the chip surface (**b**), SEM showing a close-up of temperature probes colored in green (**c**), scheme of the three probe positions on the chip on which measurements have been performed (**d**), the temperature measured at the three probe positions and DC of the applied voltage (**e**). Reproduced from [60], with permission from © 2015 AIP Publishing LLC.

Ali and teammates [57] proposed a highly sensitive humidity sensor that contained inter-digital electrodes and a graphene (G)/methyl-red (M-R) composite layer (Figure 18A). The dimensions of the electrodes are 200 μm in width (Figure 18B), 400 mm in spacing, and 400 nm in thickness. The electrode was fabricated on a polyethylene terephthalate (PET) substrate using ink-jet printing. As the sensing mechanism, the sensor electrical resistance varies inversely with the relative humidity (RH). The sensor exhibited 96.36% resistive and 2869500% capacitive sensitivity against humidity. The response and recovery times of the humidity sensor were 0.251 s and 0.35 s, respectively. This printed sensor can be easily integrated with other wearable electronic devices.

Courbat et al. [58] reported an approach used to print silver nanoparticles on paper for the fabrication of resistive temperature and capacitive humidity sensors (Figure 18C). Temperature and humidity influences on the electrical and mechanical properties of the silver structures printed on paper represent the sensing mechanism. The thickness of the printed lines is 0.8 μm, and the resistivity is 30 μΩ·cm. This sensor exhibited good linearity in the temperature range of −20 °C to 60 °C. The temperature coefficient of resistance (TCR) of the sensor was 0.0011 °C^−1^. This research offers a method that uses paper as the substrate with the inkjet printing of silver for the design of sensors.

Numerous composites composed of conductive carbon fillers have been known to display temperature dependence [147,148] and can exploited for temperature sensing [149].

Sauerbrunn and teammates introduced a “smart paint” made from latex and exfoliated graphite that was applied for continuous temperature sensing [59]. The conductive polymer composite with a nanoscale filler acts as the sensor’s core component. Conductivity variations caused by temperature changes of the substrate area affect the local current flows when a voltage is applied, leading to the potential changes of other electrodes in the sensing mechanism. The use of additive manufacturing technology made the sensors simple to integrate during fabrication. This temperature sensor can be easily mounted onto diverse existing structures and can be embedded with thermal imaging within the structures. To demonstrate continuous and distributed temperature sensing, the temperature-sensitive paint was spray-coated onto a 6 × 6 cm^2^ substrate and used in electrical impedance tomography to reconstruct a thermal image (Figure 18D).

Wickberg and teammates used three-dimensional direct laser writing to print temperature sensors based on up-conversion luminescence [60]. These micrometer-sized local temperature sensors (Figure 18E(c)) could be positioned as desired. Based on this study, a temperature accuracy of 0.5 K at a time resolution of 1 s can be easily realized. The fabrication approach for these sensors can be applied in many other situations as well.

## 4. Conclusions and Outlook

Traditional manufacturing methods have limitation of expensive apparatus, low material utilization, tedious fabrication steps and low fabrication freedom. 3D printing provides solutions to overcome all these obstacles. As the demands and application of sensing devices continue to advance, the incorporation of 3D-printing technologies into sensor elements is expected to be an ongoing trend. The use of 3D printing technologies in the manufacture of sensors has the merits of rapidly printing customized molds, high sensing sensitivity and printing accessories to fit or integrated with commercial sensors. Although 3D printed sensors have already achieved many of these advantages, traditional sensor fabrication methods are still an economical way for industrial production. Nowadays, 3D printing technology is more suitable for small batch experiments in the laboratory. The synergy of advances in sensing and 3D-printing technology offer the potential to incorporate sensors into implantable therapeutics. In the near future, a greater degree of sensitivity, throughput, and dynamic range can be achieved in a single sensor. Advancement of multi-process 3D-printing technology has the potential to realize more powerful sensors for future research and in diagnostic and therapeutic applications.

## Figures and Tables

**Table 1:** Summary of each printing method.

Technique	Principle	Material	Advantages	Limitations
Fused deposition modeling (FDM)	Extrusion-based	Thermoplastics (ABS, PLA, PC, PA, etc.); glass (new); eutectic metal; ceramics; edible material, etc.	Simple using and maintaining; easily accessible; multi-material structures; low cost	Rough surface; low resolution; high cost (for glass and metal)
Directly ink writing (DIW)	Extrusion-based	Plastics, ceramic, food, living cells, composites	Versatile	Low resolution; requires post-processing
Stereo lithography apparatus(SLA) & (Digital light procession)DLP	Photocuring	Photopolymers	High accuracy; simple	Single material; unbiocompatible
Laminated object manufacturing (LOM)	Lamination	Sheet material (paper, plastic film, metal sheets, cellulose, etc.)	Versatile; low cost; easy to fabricate large parts	Time-consuming; limited mechanical properties; low material utilization; design limitations
Selective Laser Sintering(SLS) & Selective Laser Melting(SLM)	Powder based laser curing	Powdered plastic, metal, ceramic, PC, PVC, ABS wax, acrylic styrene, etc.	High accuracy; wide adaptation of materials; high strength	Limited mechanical properties; high cost
Photopolymer Jetting(Ployjet)	Inkjet-based	Liquid photopolymers	High accuracy	High cost
3D Powder Binder Jetting (3DP)	Inkjet-based	Any material in particulate form, plaster, ceramics, sugar, etc.	No need for support material; versatile; lower cost; colorful printing	Low strength; post surface treatment; limited mechanical properties

**Table 2:** Information of 3D-printed sensors.

Application of Sensor	Method	Printer/Platform (Resolution: XY/Z (μm); Fabrication Temperature)	Material	Transduction Mechanism	3D-Printed Parts	Ref.
Strain sensors	DIW	ABG 10000, Aerotech.	Carbon-based ink	Resistance	Sensing part	[7]
	LOM		Silicone rubber	Resistance	Sensing part	[8]
	DIW	Objet Connex500 (20-85/16)	VeroBlue RGD840	Capacitance	Sensing part	[9]
	DIW		Graphene aerogel	Resistance	Sensing part	[10]
Pressure sensors	Ployjet	Objet Connex 350 (20-85/16)	TangoBlack polymer	Capacitance	Mechanical frame	[11]
	FDM	X-Truder( 230 °C)	ABS-based material	Capacitance	Sensing part	[12]
	FDM	BFB 3D Touch(200/125)	PVDF	Capacitance	Bionic sensing part	[13]
	FDM		ABS	Optical absorbance	Sensing part	[14]
Tactile sensors	FDM	Stratasys FDM Titan(-/120)	PC	Capacitance	Origami package	[15,16]
	DLP	Autodesk Ember Printer	photopolymer	Piezo resistance	Sensing part	[17]
Displacement sensors	Ployjet	DMP-3000(25-50/30)	SunTronic Jet Silver U6503	Inductance	Sensing part	[18]
	FDM		Nd-Fe-B magnets	Hall-effect	Sensing part	[19]
Accelerometers	3DP	DMP-2831(30/30)	silver nanoparticles	Capacitance	Sensing part	[20]
	FDM,SLA		Thermoplastics	Gravity	Sensing part	[21]
Angular sensors	Ployjet	DMP-3000(25-50/30)	Suntronic Jet Silver U6503	Capacitance	Electrodes	[22,23]
	FDM	Shapeways(-/16)	Plastic	Hall effect	Sensing part	[24]
Acoustics and Ultrasonics Sensors	DIW	Fab@Home	Cell-seeded hydrogel	RF reception	Bionic sensing part	[25]
	Ployjet	Objet EDEN 260V(20-85/16)	MED610 polyjet ink	Capacitance	Sensing part	[26]
	DLP		Photo-sensitive resins	Piezo resistance	Sensing part	[27]
	FDM	MakerBot Replicator2(11/100)	PLA	Frequency distribution	Sensing part	[28]
Optical sensors	Ployjet, SLA	Agilista 3000; Viper/3D systems	Photopolymer	Optical metrology	Sensing part	[29]
	Polyjet	CONNEX 350(20-85/16)	DM 8515 Grey 35 Polymer	Optical intensity	Sensing part	[30]
	3DP	Dimatix DMP 2831(30/30)	Photopolymer	Waveguides	Sensing part	[31]
	Polyjet	Objet Eden260V(20-85/16)	MED610 polymer	Light travels in straight lines	Optical fiber	[32]
Electromagnetic Sensors	DLP	Z650, ZCorp	zp^®^150	Resonance frequency	Sensing part	[33]
	FDM	ProJet HD 3000(25-50/30)	VisiJet^®^ EX 200, VisiJet^®^ S100	Inductor-capacitor (LC)	Sensing part	[34]
EEG sensors	FDM	Ultimaker 2(-/100)	PLA, ABS	Resistance	EEG electrode	[35]
	FDM	Ultimaker 2(-/100)	PLA	Resistance	EEG electrode	[36]
Magnetic field sensors	Ployjet		Nano silver or copper ink	Resistance	Sensing part	[37]
	SLA	DWS028JPlus(-/10-100)	Ferromagnetic Photopolymers	Tip deflection	Sensing part	[38]
Antennas	DIW	ABL 9000, Aerotech	Silver nanoparticle ink	RF reception	Sensing part	[39]
	FDM		Dupont 5064H	RF reception	Platform	[40]
	3DP	Self-developed	EPOLAM 5015 resin	RF reception	Platform	[41]
	SLA	Shapeways(-/16)	Steel	Patch antenna	Platform	[42]
	3D inkjet	DMP-2831(30/30)	UTDAg silver nanoink	Patch antenna	Sensing part	[43]
Biosensors	DLP	Spot-HT	Spot-A materials	Chemiluminescent	Platform	[44]
	DIW	Self-developed	PDMS, Hydrogel	Resistance	Sensing part	[45]
Chemosensors	Polyjet	EDEN260V(20-85/16)	Photosensitive resin;	Electrochemical	Platform	[46]
	Ployjet	DMP-2800(;70 °C)	Polyaniline	Electrochemical	Sensing part	[47]
	SLS	Concept Laser GmbH	Metallic particle (CL 20ES)	Potentiometric	Helical-shaped electrode	[48]
	FDM	MakerBot Replicator(11/100)	Polylactic acid (PLA)	Electro-chemiluminescence	Fluidic device and wash reservoir	[49]
Sensor for monitoring food quality	FDM	ProJet HD 3000(25-50/30)	VisiJet EX200, VisiJet S100	Electrochemical	Platform	[50]
Gas Detection Sensors	DLP	Objet Connex 500(20-85/16)	Photopolymer	Electrochemical	Platform	[51]
	Screen printing	MT650	Alumina paste	Electrochemical	Sensing part	[52]
	Polyjet	ProJet 3510 Series(30/29)	Visijet M3 crystal polymer	Resonant frequency	Separation device	[53]
Flow sensors	SLA	Self-developed	Conducting polymers	Resistance	Sensing part	[54]
	FDM	BFB 3000 (50/125)	Magnetite nanoparticle thermoplastic	Hall effect	Sensing part	[55]
Humidity sensors	Ployjet	DMP-2800	DGP-40LT-15C	Capacitance	Sensing part	[56]
	DIW	DMP-3000(25-50/30)	Graphene/methyl-red composite	Resistance	Sensing part	[57]
	Inkjet printed	DMP-2831(30/30)	DGP 40LT-15C	Resistance	Sensing part	[58]
Temperature sensors	Inkjet printed	Badger	Exfoliated graphite and latex solution	Resistance	Sensing part	[59]
	SLA	Photonics professional Nanoscribe GmbH	Photopolymer	Electro-chemiluminescence	Sensing part	[60]
